# A double-blind, randomized, parallel group study to compare the efficacy, safety and tolerability of slow-release oral morphine versus methadone in opioid-dependent in-patients willing to undergo detoxification

**DOI:** 10.1111/j.1360-0443.2009.02653.x

**Published:** 2009-09

**Authors:** Ekkehard Madlung-Kratzer, Berhard Spitzer, Renate Brosch, Dirk Dunkel, Christian Haring

**Affiliations:** 1 Psychiatric Hospital, HallTyrol, Austria; 2 Landesklinikum MostviertelAmstetten-Mauer, Austria; 3 Anton-Proksch-InstitutVienna, Austria

**Keywords:** Detoxification, methadone, opioid dependence, slow-release morphine

## Abstract

**Aims:**

Evaluation of the efficacy and safety of slow-release oral morphine (SROM) compared with methadone for detoxification from methadone and SROM maintenance treatment.

**Design:**

Randomized, double-blind, double-dummy, comparative multi-centre study with parallel groups.

**Setting:**

Three psychiatric hospitals in Austria specializing in in-patient detoxification.

**Participants:**

Male and female opioid dependents (age > 18 years) willing to undergo detoxification from maintenance therapy in order to reach abstinence.

**Interventions:**

Abstinence was reached from maintenance treatment by tapered dose reduction of either SROM or methadone over a period of 16 days.

**Measurements:**

Efficacy analyses were based on the number of patients per treatment group completing the study, as well as on the control of signs and symptoms of withdrawal [measured using Short Opioid Withdrawal Scale (SOWS)] and suppression of opiate craving. In addition, self-reported somatic and psychic symptoms (measured using Symptom Checklist SCL-90-R) were monitored.

**Findings:**

Of the 208 patients enrolled into the study, 202 were eligible for analysis (SROM: *n* = 102, methadone: *n* = 100). Completion rates were 51% in the SROM group and 49% in the methadone group [difference between groups: 2%; 95% confidence interval (CI): −12% to 16%]. The rate of discontinuation in the study was high mainly because of patients voluntarily withdrawing from treatment. No statistically significant differences between treatment groups were found in terms of signs and symptoms of opiate withdrawal, craving for opiates or self-reported symptoms. SROM and methadone were both well tolerated.

**Conclusions:**

Detoxification from maintenance treatment with tapered dose reduction of SROM is non-inferior to methadone.

## INTRODUCTION

Long-term maintenance treatment with opioid agonists is a medically significant approach for harm reduction in opioid dependence and has an important positive impact on public health [1]. The benefits of maintenance treatment are well documented, with several reports showing positive effects on indicators including mortality, physical health, risk behaviour and transmission of drug-related infectious diseases [2–5].

Although maintenance treatment is considered nowadays to be the gold standard for management of opioid dependence, detoxification is still a common and frequently demanded therapeutic modality. The primary emphasis of detoxification is not to achieve abstinence directly; rather, it may be the first step towards alternative long-term strategies [6,7]. Patients' motives for seeking detoxification treatment vary considerably, including desire to reduce illicit drug use or gain a brief respite from a life-style complicated by consumption of illicit drugs, or be the first or one of several attempts to attain a drug-free life. Importantly, detoxification may offer a period of time allowing for physical and mental recovery after uncontrolled drug use on the way to improving the patient's living conditions. Hence, detoxification can be a gateway to multiple treatment options, and can support the engagement in long-term treatment that is necessary following opioid dependence [8].

Although there are different approaches to pharmacological interventions and psychosocial therapy in the detoxification setting, overall completion rates of different detoxification regimens are generally low [6,9,10]. Tapered dose-reduction regimens over a period of 2–3 weeks are common in Austria, and are generally state-funded. During this period patients undergo supportive psychotherapy during group treatment sessions and individual social counselling. In addition, scheduled occupational therapy, physiotherapy and sports activities offer additional support during hospitalization. In Austria, more than 1000 opioid-dependent patients are referred per annum to five specialized in-patient wards—total capacity approximately 65 beds—linked primarily to psychiatric hospitals [11]. However, detoxification programmes are facing several challanges: increasing numbers of patient referrals; increasing length of waiting lists (approximately 3–4 months); and a reasonably high number of patients undergoing maintenance treatment with slow-release oral morphine (SROM).

The high proportion of patients under maintenance treatment with SROM in Austria is due to pioneering treatment programmes offering alternatives to methadone and buprenorphine, with the aim of achieving individualized treatment. The high number of SROM prescriptions can be related to the favourable side-effect profile of morphine [12,13]. Several studies have evaluated SROM as a maintenance treatment, and promising results from some of these suggest that SROM may challenge the position of methadone as the gold standard of treatment [14–17]. SROM was found to induce significantly less craving for heroin and alcohol, less depression and fewer physical complaints than methadone in these studies. Patient perceptions of improved wellbeing with SROM have led to greater acceptance by patients and a subsequent increase in its use in clinical practice. Based on this increasing patient acceptance of SROM as maintenance treatment, it is clinically important to investigate a suitable dosage regimen for SROM as an alternative to methadone for detoxification treatment. Morphine is particularly useful within opioid detoxification procedures when it is administered orally once daily in a slow-release formulation [18]. The preliminary experience of the authors of this paper with a limited number of patients confirmed the validity of this pharmacological concept. These initial findings from experienced psychiatrists were encouraging, but needed to be confirmed in a prospective, controlled trial. Here we report the first prospective, controlled clinical study comparing the efficacy, safety and tolerability of SROM versus methadone in opioid-dependent in-patients receiving opioid substitution treatment consenting to detoxification. The aim of this study was to evaluate whether detoxification with tapered dose reduction of SROM is non-inferior to methadone as measured by completion rate of the detoxification procedure.

## METHODS

### Ethical considerations

The study protocol was reviewed and approved by the ethics committee at each participating centre. The study was conducted in accordance with the Austrian Medicinal Act (BGBl 185/1983). All subjects were provided with oral and written information describing the nature and duration of the study. Written informed consent was obtained from each participant.

### Participants

This study was performed at three psychiatric hospitals in Austria specializing in in-patient detoxification treatment. All patients underwent long-term opioid substitution treatment on an out-patient basis prior to admission to the hospital. Male and female patients over the age of 18 years with a confirmed diagnosis of opioid addiction according to ICD-10 criteria, who had received maintenance treatment with either SROM (daily doses 320–960 mg) or methadone (daily doses 40–120 mg) at constant doses for ≥1 month, were eligible to enter the study. In addition, the following inclusion criteria were applied: alcohol consumption of <100 g/day during the last 4 weeks; reliable contraceptive methods (hormonal, non-hormonal) for female patients of childbearing potential. Occasional (but not daily) consumption of cocaine was acceptable. Patients were excluded from the study if they had clinically significant somatic illness (except hepatitis), acute psychotic illnesses (i.e. known schizophrenia or major depression with suicidal intent) or known contraindications to morphine or methadone. Patients were also excluded if they had received maintenance treatment with other opioids (e.g. buprenorphine, codeine derivatives) or were unwilling to follow investigator instructions. At the investigators' discretion, patients who were unsuitable to participate in the study for any other reason were also excluded, e.g. those awaiting court appearances during the following 4-week period.

### Interventions

**Figure 1:**
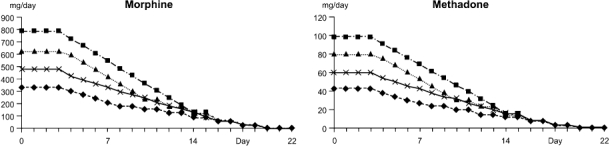
Dose reduction scheme of slow-release oral morphine (SROM) and methadone (level 1 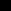, level 2 ▴, level 3 ×, level 4 ♦)

This was a multi-centre, randomized, double-blind, parallel group comparative study. After admission to hospital, patients continued with their previous maintenance treatment for 3 consecutive days on constant doses (run-in phase: days −2 to 0). During the first 2 days patients were screened under open conditions for potential participation and were assigned to one of four dose levels depending on their previous maintenance dose (level 1: SROM 960–800 mg or methadone 120–100 mg; level 2: SROM <800–630 mg or methadone <100–80 mg; level 3: SROM <630–480 mg or methadone <80–60 mg; level 4: SROM <480–320 mg or methadone <60–40 mg). After verification of the inclusion and exclusion criteria and receipt of signed consent, each subject was assigned randomly to receive either SROM or methadone according to a stratified randomization based on previous drug for maintenance treatment and dose level. Dose reduction regimens were based on a dose ratio of SROM : methadone of 8 : 1 whenever possible at each day (level 1: SROM 780 mg or methadone 98 mg; level 2: SROM 630 mg or methadone 79 mg; level 3: SROM 480 mg or methadone 60 mg; Level 4: SROM 330 mg or methadone 42 mg). These starting doses were maintained for 3 consecutive days under double-blind conditions (dose-adaptation phase: days 1–3). Thereafter, detoxification was initiated by tapered dose reductions over a period of 16 days in order to reach abstinence for 3 days (detoxification phase: days 4–22). Schedules for tapered dose reductions are presented in Fig. 1.

Study drugs were manufactured by Dr Kolassa and Merz GmbH (Vienna, Austria). For each patient an individual medication box was supplied with 22 bottles containing six identical capsules to ensure double-blind conditions (seven capsules for dose level 1 for days 1–3). Individual daily doses were prepared as capsules filled with different amounts of either SROM (morphine sulphate slow-release multi-particulate pellets: 200 mg, 120 mg and 30 mg) or methadone (methadone hydrochloride mixed with inert excipients for oral administration as solid form: 15 mg, 8 mg and 4 mg) and lactose (placebo), respectively. During the dose-adaptation phase rescue medication was made available to be taken in the late afternoon (levels 1 and 2: 120 mg SROM or 15 mg methadone; levels 3 and 4: 60 mg SROM or 8 mg methadone). The study drug was administered every morning under supervision. All comorbid diseases or medical conditions were treated in accordance with prevailing medical practice. Mirtazapine, quetiapine, topiramate, zolpidem, prothipendyl, magnesium preparations, paracetamol and naproxen were permitted as concomitant medications. Monoamine oxidase (MAO)-inhibitors, clonidine, doxepine, anti-spastic drugs, beta-blockers, lofexidine and tizanidine were not permitted. Patients with unbearable signs and symptoms of withdrawal during the detoxification phase were removed prematurely from the study.

### Objectives

The primary objective of this study was to evaluate whether detoxification with tapered dose reduction of SROM is non-inferior to methadone with tapered dose reduction over a period of 16 days followed by 3 days of abstinence, evaluated by the completion rate of the detoxification phase. Non-inferiority could be stated according to criteria outlined in the Statistical methods section. Secondary objectives were to compare the two treatments with respect to patient-reported outcomes, such as signs and symptoms of opioid withdrawal, craving for opioids and other psychoactive compounds, general wellbeing, illicit drug consumption and safety.

### Outcomes

At study entry all patients underwent a physical examination, including a full evaluation of previous and co-existing somatic and psychiatric disease and medical history. In addition, Addiction Severity Indices (ASI) were rated using the European ASI questionnaire [19]. The primary efficacy variable was study completion rate, i.e. the number of patients reaching 3 days at ‘0’ drug level after tapered dose reduction (i.e. at day 22). Secondary efficacy variables included: changes in signs and symptoms of opioid withdrawal [12-item German version of the Short Opioid Withdrawal Scale (SOWS)][20] assessed on days 0, 3, 7, 10, 14, 18 and 22 by patient self-rating; somatic and psychological symptoms [Symptom Checklist (SCL-90-R)][21] assessed on days 0, 7, 14 and 22, from which global symptom scale scores were calculated; craving for heroin, alcohol, benzodiazepines, cocaine and cannabis (rated by patients on a visual analogue scale: 0 mm = no craving, 100 mm = most intense craving) assessed on days 0, 3, 7, 10, 14, 18 and 22. All assessments were performed within 2 hours after intake of the study drug. In addition, consumption of illicit drugs was monitored. Blood samples were drawn immediately before administering the study drug on days 0, 7, 14 and 22 and analysed by tandem mass spectrometry to detect amphetamines, buprenorphine, cocaine and metabolites, benzodiazepines (flunitrazepam, lorazepam, oxazepam), ketamine,3,4methylenedioxymethamphetamine (MDMA) (‘ecstasy’), methadone, methamphetamine, monoacetylmorphine (6-MAM), morphine, phencyclidine (PCP), tetrahydrocannabinol (THC) and tramadol. Safety was assessed according to serious adverse event criteria and by recording of spontaneously reported adverse events, daily monitoring of vital signs (body weight monitored weekly), repeated physical examination and clinical laboratory data (day 0 and at study end).

### Statistical methods

All analyses were performed on an ‘intent-to-treat’ basis. All *P*-values reported are two-tailed, with *P* < 0.05 considered statistically significant. The primary efficacy variable was the completion rate in each group. To test the hypothesis of non-inferiority of the SROM group in comparison to the methadone group, the 95% confidence interval (CI) of the difference in the completion rates was calculated. Non-inferiority of the SROM group could be stated if the lower limit of the CI, for a maximum acceptable difference in completion rates of −3%, did not exceed −15%, which was defined as a clinically acceptable non-inferiority limit [22]. A sample size of 95 evaluable patients per group was estimated to be sufficient to test the non-inferiority hypothesis with a power of 80%.

Demographic and background information, as well as secondary efficacy variables, were summarized and displayed using descriptive statistical techniques. Categorical variables are presented in frequency tables, whereas continuous variables are described using mean, standard deviation and median. To explore the comparability of data between study groups, data were analysed dependent on their distribution (Mann–Whitney *U*-test; χ^2^ test). Intra-group comparisons (day 0 versus day 22) were performed using the Wilcoxon matched-pairs signed-rank test. These tests and their associated *P*-values were regarded as descriptive and not as tests of hypotheses. As there was only one primary end-point, no adjustment for multiple testing was applicable.

## RESULTS

A total of 982 patients who were hospitalized for detoxification from maintenance treatment with either SROM or methadone were screened during the study period. Of these, 600 (61%) patients did not meet the inclusion criteria and 174 (18%) refused to participate in the study.

Reasons for not meeting the inclusion criteria were: significant comorbidity (*n* = 168, 17%; maintenance dose too high or low (*n* = 107, 11%); high co-consumption of cocaine and/or alcohol (*n* = 73, 7%); slow (>4 weeks) detoxification regimen (*n* = 59, 5%); and other reasons/no information (*n* = 193, 20%).

**Figure 2:**
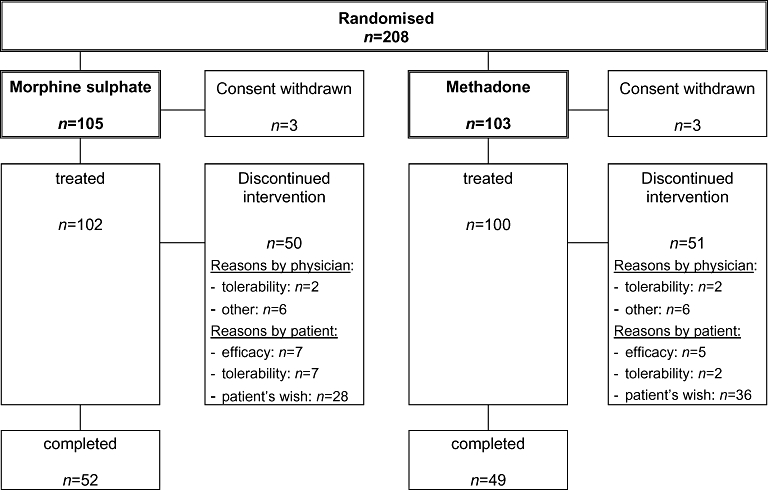
Schematic representation of patient flow throughout the study (*n* = 208)

Two hundred and eight (21%) patients were randomized into the study (117 patients at the centre in Mauer, 74 in Hall and 27 in Vienna). Six patients withdrew consent before any administration of study drug (three patients from each group). Two hundred and two patients were eligible for efficacy and safety analysis (SROM: *n* = 102; methadone: *n* = 100). Four patients in the SROM group received tramadol as concomitant medication due to withdrawal symptoms, thus violating the protocol (Fig. 2).

All patients had received previous treatment with SROM (*n* = 155, 77%) or methadone (*n* = 47, 23%) in accordance with the inclusion criteria. Treatment groups were homogeneous with respect to type (*P* = 0.450) and dose levels (*P* = 0.920) of previous treatment. At study entry, the two treatment groups were similar in demographic and disease-related variables, such as medical history. All patients had a high rate of somatic and psychiatric comorbidities typical for this patient population. The only significant difference between treatment groups in these baseline parameters was hepatitis C positivity (*P* = 0.043) (Table 1). Findings from physical examination, recording of vital functions [systolic/diastolic blood pressure, heart rate, electrocardiogram (ECG), body weight] and laboratory parameters showed no differences between treatment groups. Furthermore, patients in both treatment groups had similar ratings regarding all subdomains of the ASI illicit drug use (Table 1).

**Table 1:** Baseline characteristics and addiction history of patients.

	*SROM (n = 102)*	*Methadone (n = 100)*	*P*
Baseline characteristics			
Gender—*n* (%)			
Male	78 (76.5)	74 (74.0)	0.684
Female	24 (23.5)	26 (26.0)	
Age (years)			
Mean ± SD (median)	27.4 ± 6.2 (26.2)	28.2 ± 7.5 (26.0)	0.697
Body mass index (BMI)			
Mean ± SD (median)	23.3 ± 4.9 (22.7)	23.0 ± 4.3 (22.2)	0.865
Hepatitis B positive—*n* (%)	6 (5.9)	4 (4.0)	0.538
Hepatitis C positive—*n* (%)	29 (28.4)	42 (42.0)	0.043
Hepatitis B+C positive—*n* (%)	32 (31.3)	26 (26.0)	0.399
HIV + hepatitis B+C positive—*n* (%)	3 (2.9)	1 (1.0)	0.322
Maintenance regimen prior to study entry			
SROM—*n* (%)	76 (74.5)	79 (79.0)	0.450
Methadone—*n* (%)	26 (25.5)	21 (21.0)	
Addiction history			
Age (years) at first heroin use	17.9 ± 5.2 (17.0)	18.3 ± 5.0 (17.0)	0.325
Years of heroin use	5.2 ± 4.3 (4.0)	4.9 ± 4.4 (4.0)	0.795
Age (years) at first cocaine use	18.4 ± 6.7 (18.0)	18.4 ± 7.6 (18.0)	0.852
Age (years) at first multisubstance abuse	16.5 ± 6.5 (16.0)	17.8 ± 5.2 (17.0)	0.128
Years of multi-substance abuse	7.7 ± 6.0 (6.0)	7.9 ± 5.6 (6.0)	0.673
Number of in-patient detoxifications	1.1 ± 3.9 (0.0)	1.0 ± 2.2 (0.0)	0.581
Duration of drug-free period (months)	9.9 ± 21.9 (2.0)	5.7 ± 10.2 (1.0)	0.312

Data presented as mean ± standard deviation (SD) (median). HIV: human immunodeficiency virus; SROM: slow-release oral morphine.

**Figure 3:**
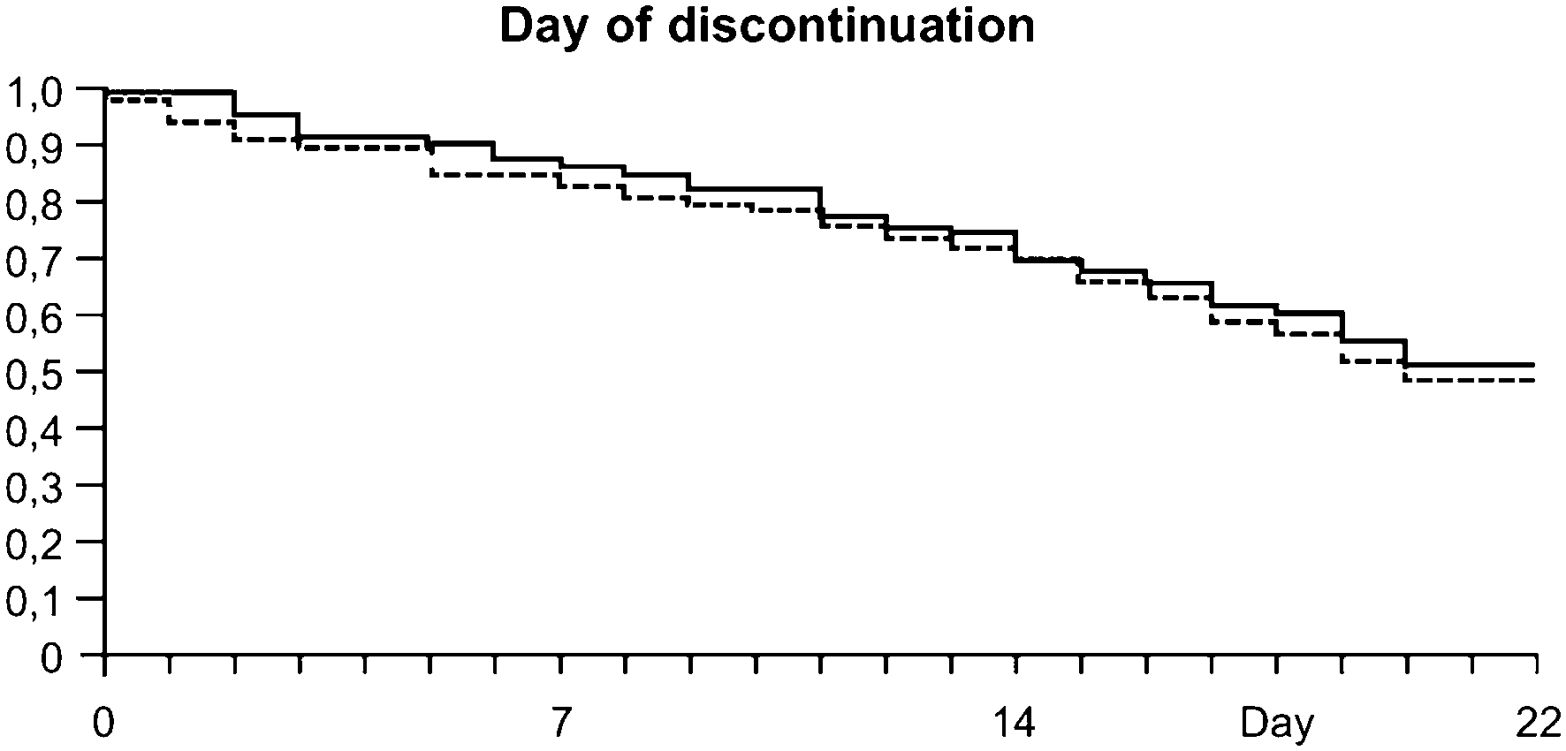
Kaplan–Meier plot of patient discontinuations during the study [slow-release oral morphine (SROM) —, methadone - - -]

Completion rate per treatment group was 51% in the SROM group and 49% in the methadone group. The difference in completion rates between SROM and methadone groups was 2% (95% CI: −12% to 16%). Therefore, according to the a priori-defined non-inferiority margin of −15%, SROM is non-inferior to methadone for detoxification (Fig. 3).

Discontinuations from the study were high in both treatment groups (SROM 49%, methadone 51%); eight patients in each group were withdrawn prematurely by the investigator. Twenty-eight (56%) patients in the SROM group and 36 (71%) in the methadone group who withdrew from the study did so voluntarily [reasons for voluntary discontinuations: left hospital without stating any reason (methadone 48%, SROM 58%), unwillingness for further detoxification (methadone 28%, SROM 28%) other reasons/no data (methadone 24%, SROM 14%)]. Neither type of previous maintenance treatment nor drug switched to for detoxification had an impact on frequency of withdrawal (χ^2^ = 0.407).

At study entry signs and symptoms of withdrawal (SOWS scores) were mild but deteriorated steadily over time (day 0 versus day 22, *P* < 0.001) (Table 2). The only difference between the groups was found on day 18 (*P* = 0.022). All symptoms showed a homogeneous pattern of changes. This was not reflected in any changes in symptoms of psychological distress based on SCL-90-R assessments.

**Table 2:** Signs and symptoms related to opioid withdrawal and psychological distress.

	*SROM Mean*± *SD (median)*			*Methadone Mean*± *SD (mMedian)*		
	*Day 0 (n*= *102)*	*Day 22 (n*= *52)*	*P*	*Day 0 (n*= *99)*	*Day 22 (n*= *50)*	*P*
SOWS	8.07 ± 6.09 (7.00)	18.32 ± 8.98 (18.00)	<0.001	8.15 ± 6.48 (7.00)	16.00 ± 7.81 (15.00)	<0.001
SCL-90-R: Global Severity Index (GSI)	0.70 ± 0.56 (0.6)	0.74 ± 0.56 (0.58)	0.326	0.77 ± 0.58 (0.61)	0.67 ± 0.51 (0.52)	0.511

SCL: Symptom Checklist; SROM: slow-release oral morphine; SOWS: Short Opioid Withdrawal Scale; SD: standard deviation.

**Figure 4:**
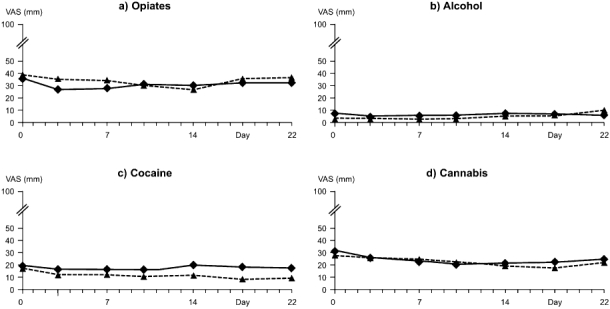
Craving for opiates, alcohol, cocaine and cannabis [self-assessment via visual analogue scale (VAS) 0 = no craving, 100 = most intense craving][slow-release oral morphine (SROM) ▴, methadone ♦]

Craving for opiates varied considerably but was generally rated as moderate. No changes became evident during the detoxification phase and there were no significant differences between groups or over time, respectively [SROM: day 0, 35.4 ± 35.1 (26.5) mm, day 22, 32.0 ± 35.1 (18.5) mm (*P* = 0.442); methadone: day 0, 38.7 ± 38.6 (27.0) mm, day 22, 36.8 ± 36.5 (25.0) mm (*P* = 0.813)] (Fig. 4a). Craving for alcohol, cocaine and cannabis was low throughout detoxification without any significant differences between groups or over time (Fig. 4b–d).

A total of 53 patients were identified via blood tests as having consumed various psychoactive substances on days 7, 14 and 22. Twenty-seven of these patients were in the SROM-group and of these, 18 completed the study and nine did not. Of the 26 patients identified in the methadone group, 17 completed and nine did not (χ^2^ = 0.922).

### Adverse events

The incidence of adverse events was low; 16 (16%) patients in the SROM group and 13 (13%) patients in the methadone group experienced at least one adverse event (χ^2^ test, *P* = 0.586). Thirty of 45 (67%) of all adverse events were rated as being unrelated, nine (20%) as possibly related (SROM: six patients; methadone: three patients) and one (2%) (methadone group) as probably related to the study drug. The majority of adverse events (23 of 45) were gastrointestinal system disorders, such as nausea (three), vomiting (10), dentalgia (five), followed by psychiatric disorders (seven of 45, e.g. dysphoria, agitation, depression, panic attacks). Two patients from the SROM group had to be transferred to another hospital ward due to comorbid medical conditions, both unrelated to study drug. Two patients had to be discontinued from study drug; one patient on day 3 due to acute hepatitis B, the other on day 21 because of acute gastroenteritis. Overall, detoxification from maintenance treatment by decreasing doses of either SROM or methadone was well tolerated.

Based on scheduled examinations, no particular changes over time or differences between treatment groups were observed during the study for physical examinations and laboratory parameters. However, heart rates increased significantly over time [SROM: day 0, 79.5 ± 12.3 beats per minute (bpm); day 22, 95.5 ± 15.9 bpm (+20%, *P* < 0.001, Wilcoxon test); methadone: day 0, 78.3 ± 9.9 bpm; day 22, 94.5 ± 15.4 bpm (+21%, *P* < 0.001, Wilcoxon test)]. Other vital functions such as diastolic/systolic blood pressure and body weight changed over time by less than 5% with no statistically significant differences between the two treatment groups.

## DISCUSSION

This is the first prospective, randomized, controlled study to evaluate the feasibility of SROM for detoxification of opioid-dependent patients from maintenance treatment. This study was powered to prove the non-inferiority of SROM to methadone. The concept of this study was based on preliminary clinical experiences with tapered dose reductions of SROM over a period of 2–3 weeks in order to reach abstinence, in accordance with the widely accepted method of managing opioid withdrawal with methadone [23–25]. Double-blind conditions were deemed necessary to minimize potential bias in patient selection and subjective assessments of efficacy and safety. Therefore, it was decided that a parallel-group study under double-blind conditions would be appropriate. In addition, the proportion of patients from each treatment group reaching a period of 3 drug-free days was defined as a suitably robust primary end-point to test the hypothesis of non-inferiority of SROM to methadone for detoxification. Frequent assessments of signs and symptoms of opioid withdrawal and craving for opiates were selected as clinically relevant secondary end-points together with self-assessments of wellbeing.

Based on the primary end-point of this study, tapered dose reductions of SROM over a 16-day period are non-inferior to methadone for keeping patients in treatment and reaching a 3-day period of abstinence independent of the starting doses. The completion rate with SROM was 2% higher than for methadone, which was within the prespecified non-inferiority margin. Previous research has shown methadone to be superior to placebo in this setting [26], and the response rate to methadone in this study was consistent with that found in other studies using similar dose-reduction regimens [27,28]. Therefore, both methadone and SROM can be viewed as superior to placebo [22]. In our study, χ^2^ tests found no impact on completion rates of switching from maintenance treatment with SROM or methadone to detoxification with either SROM or methadone, or of co-consumption. No differences were found between methadone and SROM with regard to signs and symptoms of opioid withdrawal (SOWS scores) and craving for opiates, respectively (secondary end-point). Furthermore, no differences between treatment groups were found with regard to wellbeing (SCL-90-R). Co-consumption of illicit substances was low and similar in both treatment groups. Although withdrawal symptoms increased slightly during tapering periods, withdrawal severity for both drugs were classed as ‘mild’ during these periods. There was no significant increase in subject ratings of craving throughout the study period. Tolerability of both study drugs was good. The number of patients who reported adverse events spontaneously as well as the total number of adverse events was low in both treatment groups. A trend for increasing heart rate was observed equally in both groups and can be attributed to activation of the noradrenergic pathway [29].

Although non-inferiority of SROM to methadone in a double-blind randomized study design could be demonstrated, two conspicuous features of the study warrant further discussion. First, although 208 patients were enrolled in the study, only 10% of all patients who were hospitalized for detoxification at the trial centres during the 2.5-year study period consented to participation in the study. To explain the low willingness of patients to participate in the trial, it is important to consider the circumstances of such patients at admission. Patients hospitalized at specialized detoxification wards represent a special group of opioid-dependent subjects, as they have various motives to undergo dose reduction in order to reach abstinence. They are also suffering commonly from clinically significant somatic and psychiatric comorbidities resulting in high individual stress levels and fear of detoxification [30]. The impact of double-blind study conditions also warrants consideration; patients were very anxious about the fact that neither the physician nor they themselves knew which drug was being used for detoxification.

Secondly, the completion rate and discontinuation rates observed in the present study were both approximately 50%, consistent with previous research [26,27], with discontinuations being due primarily to individual patient choice. Notably, identical discontinuation rates were recorded at all trial centres for patients treated under normal conditions not being enrolled into the study. However, these rates of premature withdrawal agree with findings in other studies and are not limited to detoxification programmes but are also a general problem in treating substance abuse [10,31].

However, premature termination of opioid detoxification is not due necessarily to a failure of treatment. As discussed earlier, the aim of detoxification treatment is not to enforce abstinence at any cost. Therefore, it is not unusual that some patients, not being able to sustain abstinence, revert immediately to maintenance treatment. However, this limited exposure to detoxification can serve as a basis for initiating mental, somatic and social improvements in an illicit drug addict's life, independent of abstinence or maintenance. The increasing number of clinical studies in both maintenance and detoxification demontrates the clinical need for individualized treatment programmes based on different medicinal products and supportive psychosocial counselling [32].

In conclusion, this prospective, double-blind, randomized study with parallel group design proved that opiate-dependent patients can be detoxified from maintenance therapy by tapered dose reductions of SROM or methadone over a period of 16 days in order to reach abstinence. SROM was shown to be non-inferior to methadone in this setting.
